# Effects of Bedding Material on Equine Lower Airway Inflammation: A Crossover Study Comparing Peat and Wood Shavings

**DOI:** 10.3389/fvets.2021.656814

**Published:** 2021-04-09

**Authors:** Jenni Mönki, Markku Saastamoinen, Ninja Karikoski, Minna Rajamäki, Marja Raekallio, Jouni Junnila, Susanna Särkijärvi, Marianna Norring, Anna Valros, Silja Oranen Ben Fatma, Anna Mykkänen

**Affiliations:** ^1^Department of Equine and Small Animal Medicine, University of Helsinki, Helsinki, Finland; ^2^Natural Resources Institute Finland, Jokioinen, Finland; ^3^Oy 4Pharma Ltd., Helsinki, Finland; ^4^Department of Production Animal Medicine, Faculty of Veterinary Medicine, University of Helsinki, Helsinki, Finland

**Keywords:** airway inflammation, equine asthma syndrome, inflammatory airway disease, horse, environmental allergens, dust, stable air quality, bronchoalveolar lavage

## Abstract

Bedding materials affect stable air hygiene, and thus the development and exacerbation of equine asthma. There is limited knowledge concerning the effects of different types of bedding material on equine lower airway inflammation. The objective of our study was to investigate the effects of bedding materials on respiratory signs, tracheal mucus score, and lower airway cytology in healthy adult horses. The study design was a prospective controlled study, and the subjects were healthy adult riding school horses (n = 32) from a single stable. Wood shavings were compared to peat, which was used as a reference bedding material. Lower airway endoscopy and sampling (tracheal wash and bronchoalveolar lavage fluid) for cytological examination were performed after each 35-day bedding period. No difference between bedding periods was observed in the respiratory rate or tracheal mucus score. Tracheal wash neutrophil percentage with the wood shavings was higher compared to the previous (*P* = 0.040) or following (*P* = 0.0045) peat period. Bronchoalveolar lavage fluid neutrophil percentage with the wood shavings was higher compared to the following peat period (*P* < 0.001). We conclude that, between the two bedding materials used in this study, peat caused less neutrophilic lower airway inflammation in horses. The information gained from this study may assist veterinarians and horse owners in selecting bedding materials, especially for horses suffering from equine asthma.

## Introduction

Equine asthma in its mild to moderate and severe forms is a frequent disease in adult horses (1, 2). Exposure to inhaled dust and molds is considered a major contributor to the development of equine asthma (1, 3) and the exacerbation of the disease (4, 5). The type of forage used greatly affects the quality of stable air, especially its dust concentrations (6–8). However, the bedding material has been shown to have a lesser, but nonetheless significant effect on stable air hygiene (8–10).

Different materials can be used for bedding in horses and all of them have advantages and disadvantages on stable air quality and animal well-being (3). Straw and wood shavings are common materials used globally in horse stables. Peat is also popular in countries where it is easily accessible and economical, such as in the Nordic and Baltic countries. Peat is an accumulation of partially decayed vegetation or organic matter, and *Sphagnum* moss is one of its most common components. It is unique to natural areas called peatlands or bogs (11).

Bedding materials create variable quantities of inhalable and respirable particles, and have different qualities regarding their abilities to absorb moisture, ammonia, and variable concentrations of microbes and endotoxins (10, 12–15). Scarce scientific literature exists concerning the effects of bedding material on equine airway inflammation (15, 16). Peat bedding is empirically considered a good choice for horses suffering from respiratory disease, but there is lack of scientific evidence supporting this (16).

The aim of our study was to investigate whether bedding material has an effect on clinical respiratory variables, tracheal mucus score, and lower respiratory tract cytology of healthy horses. We hypothesized that peat induces less airway inflammation than wood shavings. We additionally investigated the quantities of inhalable dust and ammonia in stable air with each type of bedding material.

## Materials and Methods

### Study Design

A prospective, experimental, longitudinal, controlled study was carried out from the 1st of September 2018 to the 10th of December 2018 in Ypäjä, southwestern Finland in Northern Europe. In the study, we compared the effects of two bedding materials on equine lower airway inflammation in a clinical setting, where each horse acted as its own control. Peat was considered a reference bedding material, while wood shavings were regarded as an alternative material. Each bedding material was used for 35 consecutive days in the following order: peat - wood shavings - peat.

### Animals and Management

A total of 32 adult clinically healthy research and riding school horses owned by Ypäjä Equine College and Finnish Natural Resources Institute were studied. Before the study the horses were outside on summer pasture free from ridden work. During the study the horses performed their normal riding school routines, excluding the days in which airway sampling was conducted. The horses were housed indoors for ~18 h per day and ridden in an indoor riding arena for 2–3 h/day. The remaining 3–4 h of the day were spent outside on sand paddocks.

**Figure 1:**
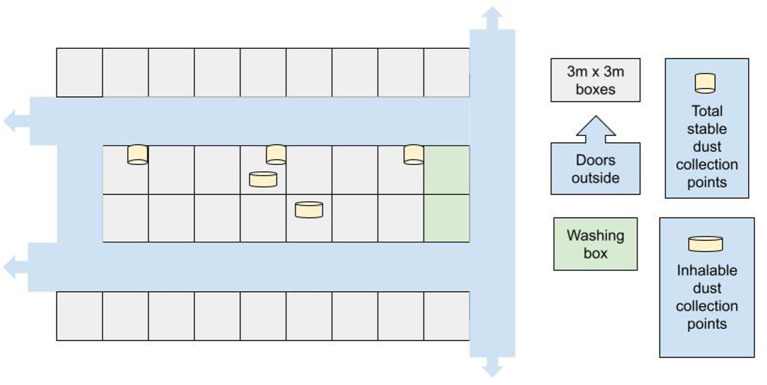
Outline of the stable.

The horses were housed in a stable in individual boxes (3 × 3 m), with box doors opening into common indoor corridors (width 2.8 m) (Figure 1). Stable room height was 3.5 m and the boxes were situated along two parallel aisles with a common airspace. Ventilation of the stable was mechanically enforced with an extractor technique, and the stable doors were kept open as much as possible during the daytime, depending on weather conditions. The stables were manually cleaned on a daily basis, mainly when the horses were outside. All feces and wet materials were removed from the box, and a similar quantity of new bedding material was added to maintain an approximately bedding depth of 10 cm. An average 77 L of peat and 83 L of wood shavings per box were added per day. This stable had used peat as a bedding material for years.

The horses were fed haylage (dry matter 73%) wrapped into large round bales using stretch film and pelleted compound feed (dry matter 88%) from the same provider and manufacturer over the entire study period. The haylage fulfilled the criteria for good-quality haylage (17). It was fed from the box floor thrice per day: at ~6 a.m., noon, and 6 p.m. Each horse consumed approximately 8–10 kg of haylage and 1–2.5 kg of pelleted feed per day based on their individual needs. The clinical status of the horses was monitored on a daily basis by the stable staff and equine college students working with the horses, who recorded any signs of cough and/or nasal discharge.

### Bedding Materials

Bedding materials were stored in a barn separate from the main stable during the experiment. The *Sphagnum* peat (40% moisture, pH 4.0–4.8) used in this study was manufactured for horse bedding (Vapo Group, Vantaa, Finland). It was packed into plastic-covered 85-L bales. Wood shavings were used as an alternative bedding material. Wood shavings were made of Finnish coniferous wood (Fortum HorsePower, Espoo, Finland) and fine aggregate was removed during the manufacturing process. Wood shavings were packed into plastic-covered 20-kg bales.

### Monitoring Stable Air Quality

The quantity of inhalable dust and the ammonia concentrations were measured in two boxes, one in each stable corridor, for both bedding materials (Figure 1). The measurements were carried out during two consecutive days at the end of the shavings period and the second peat period, when environmental conditions were considered to have stabilized.

The inhalable dust was collected by applying a standard method (18). The air was collected with a 25-mm diameter filter on an IOM sampler using a sampling pump (Gilian 5000, Sensidyne/Schauenburg Group, Germany). The laboratory of the Finnish Institute of Occupational Health (TTL, Helsinki, Finland) provided the equipment, which was calibrated before and after each 2-day measuring period using a reference meter (Mesalabs Defender 510, Mesa laboratories, USA). The sampler was attached to the partition wall inside the box at a height of 1.5, 1.0 m from the box front wall. The air sampling volumetric flow through the pump was 2.0 L/min and the sampler collected the inhalable dust, i.e., particles < 100 μm. The average air quantity of the sampling was 954 L (SD 6.6).

The air samples were collected during three 8-h periods: 6 a.m.−2 p.m., 2 p.m.−10 p.m., and 10 p.m.−6 a.m. The first measuring period covered morning and midday feeding along with the time spent cleaning the boxes and adding new beddings. During the second period, the horses were moved to the paddocks or riding lessons and received their evening meals. During the third period, no activities were carried out in the stable, and the horses remained in their own boxes. The inhalable dust (convention) load was analyzed gravimetrically (applying standards EN 481:1993 and ISO 7708:1995), i.e., weighing the whole IOM sampling cassettes, and the results are presented in mg/m^3^ (18). The detection limit was 0.1 mg/m^3^. Total stable dust was additionally measured by collecting dust with open plastic containers (13-cm deep containers, surface area 188.8 cm^2^) placed at a height of 2.5 m at three locations within the stable (Figure 1). The quantity of dust collected in the containers was weighed and expressed as mg/day.

Stable air ammonia concentrations (ppm) were measured using an Accuro gas detection pump that draws air through sampling tubes (Dräger Safety AG, Lübeck, Germany) with a similar schedule and from the same locations as the inhalable dust sampling. The measurements were performed at 5.45 am, when all doors and windows had remained closed for the night and before the beginning of any activities in the stable. The measuring height was 1.2 m from the box floor. Stable temperatures (°C) and air humidity (%) were measured daily at 6 am from both corridors. The monthly means of the local outdoor temperatures during the study were obtained from the Finnish Meteorological Institute.

### Airway Sampling: Schedule and Procedures

On days 34 or 35 of each bedding period all the horses were examined and samples were obtained. The examination included a clinical examination with cardiothoracic auscultation, measurement of heart rate and respiratory rate, measurement of rectal temperature, palpation of submandibular lymph nodes, and inspection for the presence of nasal secretions. Horses were examined and sampled in their own boxes.

After clinical examination, the animals were sedated with intravenous detomidine (0.006–0.02 mg/kg, Domosedan vet inj 10 mg/mL, Orion Corporation, Espoo, Finland) and butorphanol (0.006–0.02 mg/kg, Butordol 10 mg/mL inj, Intervet International, Boxmeer, Netherlands). The airways were examined with a fiber-optic video endoscope (Pentax EG-2940K, 10 mm × 100 cm), and the clinical findings were recorded by a single experienced person (JM) using a tracheal mucus score from 0 to 5 (19).

Subsequently tracheal wash (TW) aspirate samples were obtained with a dispensable single lumen catheter (2.3 mm × 220 cm Equivet Endoscope flushing catheter, Kruuse) via an endoscope working channel using 20 mL of saline.

Following the endoscopy, bronchoalveolar lavage fluid (BALF) samples were obtained using a soft rubber tube (10 mm x 240 cm Equivet B.A.L. catheter with a balloon (10 mL), Kruuse) using a blind technique. Local anesthesia (40 mL of 1% lidocaine diluted with saline, Lidocain 20 mg/mL inj, Orion Corporation, Espoo, Finland) was administered prior to 300 mL of physiological saline (room temperature), which was injected in one single volume followed by immediate manual aspiration. Samples were considered adequate when a foamy surfactant layer was detected. The BALF samples were immediately placed on iced water and submitted to laboratory processing within 1 h.

### Sample Processing and Analyses

Tracheal wash samples were prepared for differential cell counts by centrifugation and subsequent smearing of the cell pellet onto a slide. Pooled BALF samples were filtered through a single-layer cotton gauze and the fluid volume was recorded. The BALF cell count was determined using trypan blue stain (1:1), after which the sample was cytocentrifuged (Thermo Scientific Cytospin 4 centrifuge; Thermo Fisher Scientific, Waltham, MA, USA). All slides were stained with May-Grünwald-Giemsa stain.

A single blinded experienced observer counted 300 cells for TW and 400 cells for BALF under light microscopy at 400x magnification and also performed differential counts for macrophages, lymphocytes, neutrophils, eosinophils, mast cells, and epithelial cells. The results were expressed as percentages of the total cells.

### Statistical Analyses

The sample size was calculated (http://nrs.harvard.edu/urn-3:HUL.InstRepos:8160851) with BALF neutrophil percentage as a primary outcome in a crossover design (2-tailed, power 0.8, significance 0.05, standard deviation 2, estimated difference in means 2, estimated group size 20 horses). The remaining statistical analyses were performed using the IBM SPSS statistics system for Windows.

If a certain cytology variable was unmeasurably low, the value was imputed as half of the lowest measured proportion. The Shapiro-Wilks test was used to evaluate the normality of the data distributions. Standard data transformations (log, square root) were used to normalize the distributions of the cytology variables. The differences between the periods in the clinical and cytology variables were analyzed with a linear mixed-effects model, where period was used as the sole fixed effect and horse as the random effect. Pairwise differences (with 95% CI) were estimated from the models. Values were considered significant at *P* < 0.05.

## Results

### Animals

Thirty-two adult (mean age 11.8 years, range 4–18) healthy riding school horses were included in the study. Two horses were accidentally sold after the first peat period, and they were replaced by two new horses for the remaining study period (wood shavings and peat following wood shavings). The change in individuals was taken into account when performing paired testing in the statistical analyses. The final composition of the study population was 19 Finnhorses and 13 Warmbloods, with an equal number of mares (*n* = 16) and geldings (*n* = 16). All horses remained free from signs of respiratory disease, including cough, during the entire study period.

### Complications

During the sampling after the second peat period, one horse, a 16-year-old Warmblood mare, began bleeding from the lower airways while aspirating BALF. This was observed as the fluid turned sanguineous, and BALF suction was stopped immediately. The mare did not show signs of epistaxis or external signs of respiratory distress and was closely monitored afterwards. However, on the following day, this subject developed fever and was started on oral antibiotics, after which it made a full recovery in 1 week. Due to this complication, this horse was excluded from the second peat period of the study.

### Tracheal Mucus Score and Respiratory Rate

No statistical difference between periods was observed in tracheal mucus scores or respiratory rates (Table 1a).

**Table 1a:** Cytology results [tracheal wash (TW), bronchoalveolar lavage fluid (BALF)] and respiratory parameters of 32 horses housed on peat or wood shavings.

	**Peat 1**	**Wood shavings**	**Peat 2**
**TW cell type %**			
Macrophages	66 (0–95.7)	58.7 (5–94.4)	71.3 (15-98)
Lymphocytes	0.7 (0–5.7)	1 (0–5.7)	0.7 (0–2.7)
Mast cells	0 (0–0.4)	0 (0–0.7)	0 (0–0.7)
Epithelial cells	7.4 (0.7–100)	2 (0–37.7)	8.6 (1–82.4)
**BALF cell type %**			
Macrophages	38.8 (26–64.8)	41.7 (18.5–64.5)	44.5 (27–69.8)
Lymphocytes	53 (5.8–69.8)	52.5 (28.3–65.5)	52.8 (25.5–71.8)
Epithelial cells	0 (0–11.3)	0 (0–0.5)	0 (0–0.3)
BALF total nucleated cell count (cells/μL)	271 (110–860)	288 (50–610)	224 (70–380)
Respiratory rate (breaths/min)	12 (9-20)	12 (8-24)	12 (8-20)
Mucus score	0 (0–2)	0 (0–2)	0 (0–2)

*Numeric values are presented as means with ranges in brackets*.

### Cytology

**Figure 2:**
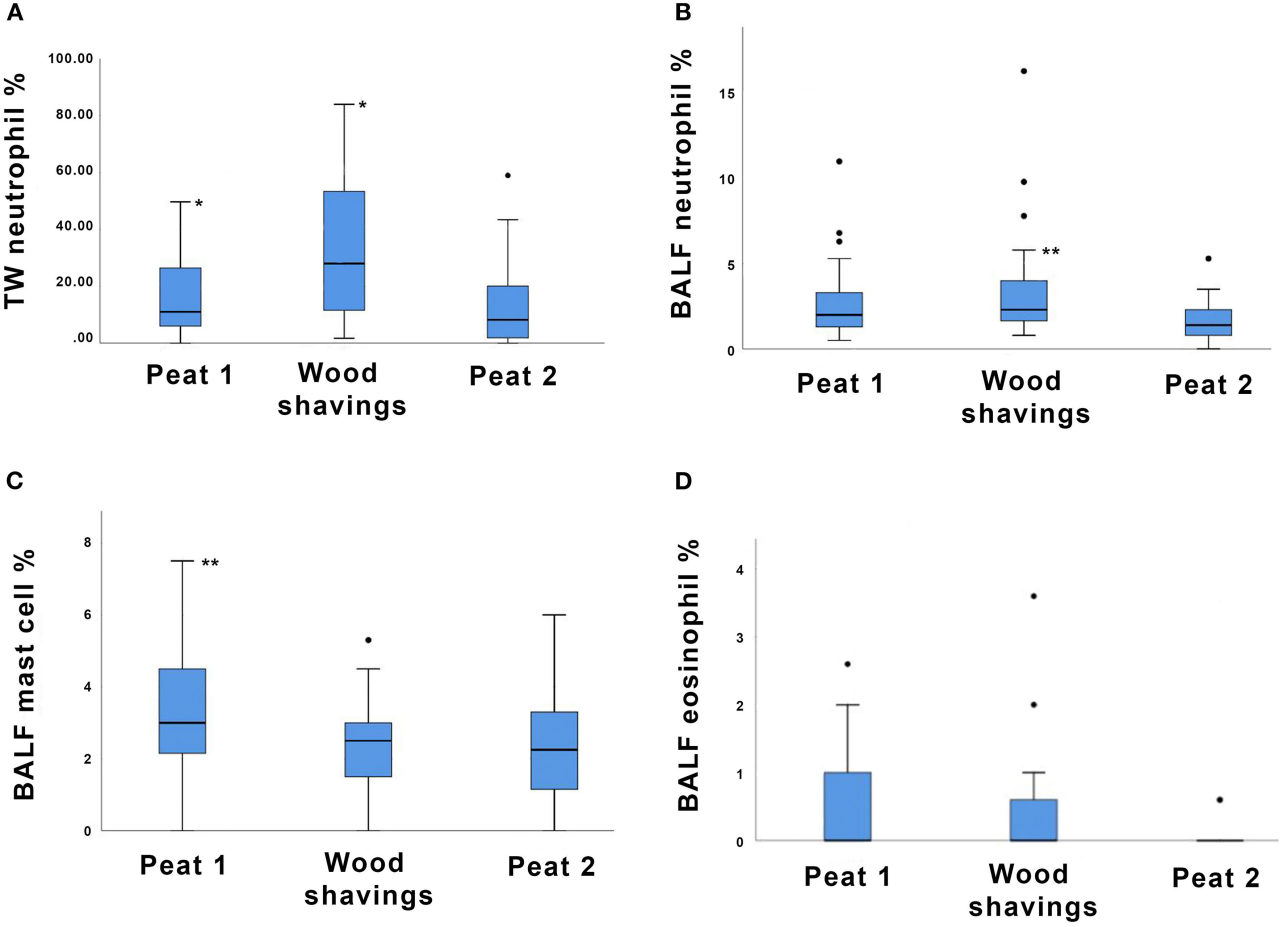
Box plot depicting **(A)** tracheal wash (TW) neutrophil percentages, **(B)** bronchoalveolar lavage fluid (BALF) neutrophil percentages, **(C)** BALF mast cell percentages, and **(D)** BALF eosinophil percentages of 32 horses housed on different bedding materials after each bedding material (Peat 1, Wood shavings, Peat 2). Each box represents the IQR (i.e., 25th to 75th percentiles), the horizontal line in each box represents the median, the whiskers represent the range. Significant differences between groups are indicated as follows: in **(A)** * indicates *P* < 0.05; in **(B,C)** ** indicates *P* < 0.001.

The tracheal wash neutrophil percentage during the wood shavings- period was higher compared to the peat- period both before (*P* = 0.04) and after (*P* = 0.0045) the wood shavings- period (Figure 2A).

The BALF neutrophil percentage was higher during the wood shavings- period compared to peat- period after the wood shavings- period (*P* < 0.001, Figure 2B). The BALF neutrophil percentage of the wood shavings- period did not differ from the peat- period before the wood shavings- period. The BALF mast cell percentage was lower during the wood shavings- period compared to the previous peat- period (*P* < 0.001, Figure 2C). For the odds of detecting a measurable BALF eosinophil percentage, a trend was evident for a higher eosinophil percentage during the wood shavings- period compared to peat- period after the wood shavings- period (*P* = 0.055, Figure 2D). The proportions of the remaining TW and BALF cell types are presented in Table 1a.

### Stable Air Quality

The average indoor temperatures and humidity percentages, along with outdoor temperatures, are presented in Table 1b. The inhalable dust quantity was under the detection limit (<0.1 mg/m^3^) for most of the time. The detection limit was exceeded during one sampling period for both beddings – this was during mocking and when new bedding materials were added, when the horses were not inside the stable. The average quantities of inhalable dust for each bedding material are presented in Table 1b as means of the two corridors for inhalable dust and as means of stable dust. Stable air ammonia concentrations were low for both bedding materials (Table 1b).

**Table 1b:** Summary of stable air quality during different bedding periods.

	**Peat 1**	**Wood shavings**	**Peat 2**
Respirable dust (mg/m^3^)	—	<0.10 (<0.10–0.11)	<0.10 (<0.10–0.13)
Total stable dust (mg/day)	2.8 (1.7–4.2)	3 (2.7–3.3)	5.7 (2.3–10.3)
Amount of ammonia in stable air (ppm)	—	0.4 (0.25–0.6)	0.0 (0.0–0.0)
Indoor temperature (°C)	14.0 (12-19)	11.0 (9-15)	11.0 (7-13)
Indoor air humidity (%)	77.8 (65–90)	80.8 (60–91)	86.2 (75–94)
Outdoor temperature (°C)	10.9 (4.1–18.3)	5.3 (-3.2–13.1)	4.2 (-5.9–7.9)

*Numeric values are presented as medians with ranges in brackets*.

## Discussion

This is the first study that compares the effects of peat and wood shavings as bedding materials on the lower airway cytology of healthy horses. There is limited previous research on peat as a horse bedding material, despite it being popular for this usage for decades in certain countries. In this study, higher percentages of neutrophils in lower airway samples were detected when horses were kept on wood shavings compared to peat. This finding is in line with the study of Saastamoinen et al. (16), who found increased tracheal mucus scores in horses kept on wood shavings compared to peat. In the present study, an increase in neutrophils was detected in both tracheal wash (TW) and bronchoalveolar lavage fluid (BALF), indicating that an increase in neutrophils also occured in the bronchoalveolar level. In TW the median neutrophil percentage after the wood shavings period exceeded the upper reference range for this cell type [<20% (2)] (Figure 2A). In BALF the median neutrophil percentage remained within normal limits [<5% (2)] after the wood shavings period despite the statistically significant change compared to sampling after the second peat period (Figure 2B). The horses used in this study were healthy adults and none suffered from any form of equine asthma based on their histories, thus neither clinical respiratory signs nor severe inflammatory reaction of the airways were expected. Despite changes in airway neutrophilia, all horses remained clinically asymptomatic for the entire study period. In previous studies (3), lower airway neutrophilia in asymptomatic horses has been linked to increased dust exposure.

This study showed a trend for BALF eosinophil percentage to increase during exposure to wood shavings compared to the peat- period after wood shavings. On the other hand, the mast cell percentage was lower during the wood shavings- period compared to the previous peat- period. Both eosinophils and mast cells have been associated with allergy and airway hyperresponsiveness, and increased quantities of either cell type in BALF may be used to diagnose certain asthma subtypes in horses (2). In our study, an increase in eosinophil percentage was accompanied by a significant decrease in mast cells, indicating that the mechanism causing the shift was different for these two cell types. Interestingly, some evidence also suggests that high respirable particulate exposure correlates with elevated eosinophil and mast cell percentages in BALF, rather than with neutrophilia (3). Unfortunately, our study design did not allow studying the effect of particulate exposure on various cell types.

Most of the previous research has focused on comparing the particulate exposure levels caused by different bedding materials rather than on the clinical effects of bedding materials on airway inflammation (3). Furthermore, the research on stable air quality has focused on rather extreme conditions, e.g., comparing high dust-producing combinations, such as straw and hay, to the other extreme of a low dust-producing combination such as wood shavings and haylage (7, 20, 21). On the other hand, information regarding stable conditions, such as air humidity, temperature, and gas concentrations, is lacking in many studies focusing on equine respiratory health.

The methods for analyzing stable air quality, especially dust concentrations, vary widely between studies, making result comparisons somehow challenging. In addition, the behavior of individual horses in the boxes may have a remarkable yet poorly studied effect on the quantity of inhaled respirable particles, thus increasing data variability (22). Ward et al. (9) showed wood shavings to be superior compared to pelleted newspaper or straw bedding regarding respirable dust levels in stable air. On the other hand, the use of straw pellets was associated with lowest particle concentrations (particle size larger than 10 μm) compared to wood shavings and straw (10). Forage type is a major contributing factor to respirable dust in the breathing zone of the horses (7). However, in the present study, forage and feeding routines remained the same throughout the study period, minimizing the effect of feeding on the results.

Higher respirable dust concentrations have previously been shown to correlate with the degree of airway inflammation (23, 24). Bedding material also affects other factors besides dust in the stable air, such as endotoxin, 1,3-β-glucan, and ammonia concentrations (6, 20). These factors may also contribute to the inflammatory cascade in equine lower airways. Therefore, the causal relation that bedding material causes more dust in stable air and subsequently more airway inflammation may be an overly simplified conclusion. In a previous study with a small number of horses, an increase in the tracheal mucus was observed when shavings was compared to peat bedding, while no difference could be detected in the quantity of dust in stable air (16). Unfortunately, the dust measurements performed in our study were not enough to be used for statistical analyses. However, as seen in Table 1b, no distinct differences could be observed in the quantities of total stable dust or inhalable dust that would explain the difference it can be seen in the cytology results. Therefore, other factors apart for the dust quantity are likely to contribute to the changes in lower airway neutrophilia observed in the present study.

The less airway inflammation induced by peat bedding compared with wood shavings may be explained by dust particle size. Although a seemingly large quantity of dark macroscopic dust can be seen covering all surfaces in a peat-bedded stable, alveolar-level contamination most likely remains low due to the large particle size of peat. Tissari et al. (25) investigated the fine particle emission at the site of milled peat production and found that major particle mass emission was attributed to particle sizes larger than 10 during most of the process steps. The smaller the particles, the more detrimental they are to the airways (26). Particles with a diameter larger than 10 μm are large enough to settle quickly under the influence of gravity and are thus deposited in the proximal airways (3). Inhalable dust, i.e., particles <100 μm, were collected by a sampler in our study, and assessing the proportion of small particle-sized dust was not possible. The collectors were placed on the wall of each box at a height of 1.5 m, and the samples were therefore collected from the ambient air in the horse's stall and not from the breathing zone of the horse.

Although the stable used in our study was mechanically ventilated and professionally managed, some day-to-day variation likely occurred in the ventilation. For example, the changing weather conditions had a non-measured effect on how long the barn doors were kept open during the daytime. The effect of wind on the air dustiness is minimal in the region where the stable was located.

The average indoor temperatures remained within the target temperature range (8–12°C) for horse stables in Finland (27), but the stable air humidity was much higher compared to the target value (50–55%) for all investigated periods (Table 1b). The presence of airborne dust particles is influenced by temperature and air humidity. As the weather in Northern Europe is quite different from that of southern countries, the results of our study may not necessarily be applicable to more temperate regions of the world.

Peat has a good capacity to bind ammonia, a feature based on its naturally low pH value (28). However, in our study, ammonia levels in the stable air remained low with both peat and wood shavings.

Despite this and previous studies observing certain beneficial qualities of peat (16), the use of peat as a horse bedding material raises currently unanswered questions. As an organic material manufactured via various processes, the hygienic quality and dustiness of peat may be somewhat unpredictable (12). In one study, a mixture of peat and wood shavings resulted in greater bacterial air contamination compared to straw or crushed wood pellet bedding (15). Peat may contain high numbers of mycobacteria (29), but an association of peat and mycobacterial infections in horses has not been established (30). The environmental sustainability in peat harvesting has to be taken into consideration, as the peatland ecosystem is an efficient carbon sink and an extremely slowly renewable energy source (11).

Airway neutrophilia caused by exogenous triggers is evident in asthmatic horses 6 h after exposure (31) but may persist for some weeks after a change of environment and remission of clinical signs (32). For practical reasons, each bedding material period in the present study consisted of 35 days. However, longer exposure may induce more marked differences between the bedding materials. In addition, as the periods were short, a carry-over effect may have been produced by a previous period causing bias to the results of the following period. Our study design (peat—wood shavings—peat) partly compensates for this bias. The first airway sampling was done only after the first peat period and not before the initiation of the study when the horses were stabled after the summer. If some horses were affected by summer pasture- associated asthma, this could have caused some carry-over effect in the neutrophil proportions of the first samples.

## Conclusions

In conclusion, compared to wood shavings, peat bedding caused less neutrophil percentage in the lower airway of healthy horses. The results of this study support the initial hypotheses of peat being a suitable option for horse stable bedding material and superior to wood shavings when considering equine airway health. The effect of forage on the quantity of respired particles in horses is well-established. This information has made haylage or even pelleted food a popular choice of roughage for horses suffering from asthma, while advice concerning beneficial bedding materials remained scarce. The information gained from this study may assist veterinarians and horse owners in selecting bedding materials, especially for horses suffering from respiratory diseases. Nevertheless, only clinically healthy horses were used in this study, and more research on the effect of bedding materials in horses with different respiratory conditions are warranted.

## Data Availability Statement

The raw data supporting the conclusions of this article will be made available by the authors, without undue reservation.

## Ethics Statement

The animal study was reviewed and approved by the Finnish National Experimental Animal Committee. Written informed consent was obtained from the owners for the participation of their animals in this study.

## Author Contributions

JM, MS, and AM contributed to the following steps of the manuscript preparation process: generating the hypothesis and designing the experiment, organizing and conducting the experiment, interpreting and analyzing the results, and writing and revising the manuscript. NK contributed to generating the hypothesis, designing the experiment, and organizing and conducting the experiment. JJ contributed to designing the experiment and interpreting and analyzing the results. MRaj contributed to generating the hypothesis and designing the experiment. MRae contributed to organizing and conducting the experiment. SS and MN contributed to organizing and conducting the experiment and interpreting and analyzing the results. AV contributed to generating the hypothesis and designing the experiment. SO contributed to conducting the experiment. All authors contributed to the article and approved the submitted version.

## Conflict of Interest

JJ was employed by the company 4Pharma Ltd. The remaining authors declare that the research was conducted in the absence of any commercial or financial relationships that could be construed as a potential conflict of interest.
